# The Potential of Digital Phenotyping and Mobile Sensing for Psycho-Diagnostics of Internet Use Disorders

**DOI:** 10.1007/s40429-021-00376-6

**Published:** 2021-07-08

**Authors:** Christian Montag, Hans-Jürgen Rumpf

**Affiliations:** 1 Faculty of Engineering, Computer Science and Psychology, Department of Molecular Psychology, Institute of Psychology and Education, Ulm University, Helmholtzstr. 8/1, 89081 Ulm, Germany; 2 Department of Psychiatry and Psychotherapy, University of Lübeck, Lübeck, Germany

**Keywords:** Internet Use Disorders, Problematic Internet use, Internet addiction, Smartphone addiction, Social media addiction, Digital phenotyping, Mobile sensing, Psychoinformatics, Big Data

## Abstract

**Purpose of Review:**

The present paper provides an accessible overview on the potential of *digital phenotyping* and *mobile sensing* not only shedding light on the nature of Internet Use Disorders (IUD), but also to provide new ideas on how to improve psycho-diagnostics of mental processes linked to IUD.

**Recent Findings:**

In detail, the psycho-diagnostic areas of prevention, treatment, and aftercare in the realm of IUDs are focused upon in this work. Before each of these areas is presented in more specificity, the terms *digital phenotyping* and *mobile sensing* are introduced against the background of an interdisciplinary research endeavor called *Psychoinformatics.* Obstacles to overcome problems in this emerging research endeavor—sensing psychological traits/states from digital footprints—are discussed together with risks and chances, which arise from the administration of online-tracking technologies in the field of IUDs.

**Summary:**

Given the limited validity and reliability of traditional assessment via questionnaires or diagnostic interviews with respect to recall bias and tendencies to answer towards social desirability, *digital phenotyping* and *mobile sensing* offer a novel approach overcoming recall bias and other limitations of usual assessment approaches. This will not only set new standards in precisely mapping behavior, but it will also offer scientists and practitioners opportunities to detect risky Internet use patterns in a timely manner and to establish tailored feedback as a means of intervention.

## Background

At the moment of writing, more than 60% of the world’s population have access to the Internet [1]. Since the introduction of the iPhone in 2007, the distribution of the smartphone has seen rapid progress resulting in more than six billion smartphone subscriptions in 2020 [2]. These numbers also reflect the development towards ubiquitously available mobile Internet access. With the rise of the smartphone, also a rapid gain of social media users could be observed. Currently, an impressive number of nearly four billion social media users has been estimated [3].

Without doubt the worldwide movement towards digital societies led to much improvement on many levels: For instance, it has become very convenient for humans to communicate via far distances at low cost or to search for information from nearly everywhere as long as a signal is available. Moreover, online users have the chance to express themselves via social media or search their way in unknown territory by means of online map services.

Despite the many positive outcomes of digitalization, scientists more and more discuss also the negative aspects of Internet use. Among others, the rapid development towards a digital society is accompanied by a growing number of humans developing addictive behaviors to the Internet. However, due to imprecise measurements, lack of clear definitions, and representative samples, prevalence estimations have to be regarded as insufficient and likely to overestimate the occurrence of IUD [4]. In the absence of clinically valid thresholds, data-driven latent profile analyses result in lower estimates [5]. In this context, also a study by Stevens et al. [6] is noteworthy showing that a focus on functional impairment when establishing prevalence rates for Gaming Disorder (a distinct IUD) also led to lower prevalence rates.

In 1996, Kimberly Young [7] already described the case of a person potentially being addicted to the Internet. More than 20 years after this initial report, Internet Use Disorders are discussed in several domains including the excessive use of online platforms linked to gaming, gambling, shopping, social media, and pornography [8]. The ICD-11 issued by the World Health Organization (WHO) officially recognizes problem behaviors related to gaming and gambling. Whereas pathological gambling (now “Gambling Disorder”) has been already included in 1980 in DSM-III (then in the category of impulse control disorders) [9], Gaming Disorder currently represents the first disorder being officially recognized and linked to problematic online behavior (6C51.0, predominantly online variant) [10, 11] based on sufficient evidence and clinical needs [12, 13]. Gaming Disorder has been ratified to be included in ICD-11 in May 2019. Please note that although the “disorder-terminology” is used to characterize problematic gaming/gambling by the WHO, the category among which both Gambling and Gaming Disorder can be found in ICD-11 is called “Disorders due addictive behaviours”^1^. This fact hints towards the similarity of problem gaming/gambling behavior with other addictive disorders—at least from the perspective of the WHO. But it is also true that this debate has not been settled on the side of many scientists and led to protests both from the gaming industry [14] and also from several scholars [15, 16]. Please note that beyond Gaming Disorder/Gambling Disorder, currently no other areas of digital overuse are officially recognized in the manuals of the World Health Organization (WHO) or the American Psychiatric Association (APA). Therefore, currently, also topics such as overuse of social media or the Internet in general are still objects of controversial discussions (e.g., see also discussion on social media use and well-being [17]), with some prominent theories rather seeing overuse of the Internet as coping behavior to deal with adversities in everyday life (for instance, see the conceptual framework proposed by Kardefelt-Winther [18]). The controversy around technological use disorders also mirrors in the debate on how to correctly name phenomena such as excessive social media use (e.g., social media addiction, problematic social media use, social networks use disorder).

In the following, we will use the term Internet Use Disorders (IUD) as an umbrella term for the different forms of overuse of online content (together with the disorder term in distinct problem online overuse areas). For us, it is important to explicitly state that we use these terms (a) to strive for unification of terminology in the literature and (b) to align the terminology with those proposed by the WHO in the gaming and gambling area. We are aware of the importance to not over-pathologize everyday life behavior [19] and explicitly mention that we do not want to do so in this article by using the disorder term in areas which have been not officially recognized so far. Aside from our view point, a recent opinion piece correctly mentioned the importance to apply fairness at how to currently label the phenomenon at hand [20]. Of note, a recent other opinion paper also mentioned three criteria to be fulfilled to speak of an addictive behavior, namely the clinical relevance of the behavior under investigation must be established, a theoretical fit with an addiction framework should be apparent, and finally empirical evidence supporting these ideas at best following multi-methodological approaches need to be observed [21]. For an up-to-date theoretical framework to understand IUDs, please see the I-PACE model [22, 23].

## Digital Phenotyping and Mobile Sensing as New Tools to Gain Insights Into Internet Use Disorders

The psychological and psychiatric sciences currently are seeing a paradigm shift. With the rise of the Internet of Things, many scientists argue that it is high time to more consider the study of digital footprints to improve psychodiagnostics, but also to enhance the treatment and aftercare of mental disorders [24•]. Already in 2012, Miller [25•] published the “smartphone psychology manifesto” to hint towards the potential of using the smartphone technology to ameliorate psychological research. With about 50% of human mankind having access to the smartphone technology [2], it is natural for scientists to also rely on the smartphone as an important data source. Many smartphone users carry this device around 24/7. The vision behind implementing smartphones in psychological and psychiatric research is much broader though than “just” relying on the smartphone as relevant data source. By background-online-tracking and analysis of human-smartphone-interaction, several scientists foresee the rise of a new discipline called *Psychoinformatics* [24•, 26], where methods from the computer sciences can aid the psychological/psychiatric sciences to deal with the manifold problems coming with self-report inventories or well-controlled experiments. Among others and regarding the self-report, problems such as tendencies to answer in a social desirably way, recall bias, or lacking introspection skills are discussed to reduce the validity and reliability of such data. In the realm of psychological experimental works, it is clear that confounding variables can be well controlled, but the experiment is very costly to conduct and usually limited with respect to the number of humans which can be tested [27]. Moreover, it is questionable if data from such an artificial setting can be transferred one on one to everyday life contexts (hence, the ecological validity is questioned). Inclusion of the study of digital footprints including tracking technologies based on smartphones could therefore represent a much welcomed addition to the toolbox of many scientists. *Psychoinformatics* likely enables researchers to more easily record how humans behave in everyday life and to do this on a longitudinal level. These opportunities are supplementary to the principles of Ecological Momentary Assessment (EMA) that has been introduced as a novel assessment approach in real-life settings and has, for example, been applied to tobacco smoking [28]; however, methods from *Psychoinformatics* go beyond self-report. In addition, both approaches can be combined fruitfully.

In the *Psychoinformatics* literature, one easily stumbles upon the terms *digital phenotyping* and *mobile sensing* [29]. *Digital phenotyping* describes the ability to infer psychological traits/states from digital footprints arising from the manifold sources of the Internet of Things where everything from the coffee machine to the car is connected to the Internet [30] (for a good overview, see also the work by Insel [31••]). Please note that from onboard diagnostics in cars, already the gender of a person can be predicted [32], from Facebook-Likes personality traits [33] or from variables such as the tracked shower behavior via smartcards the academic performance of person groups [34]. *Mobile sensing* describes to sense a person’s psychological traits/state from mobile devices such as the smartphone. Hence, it is a closely related term to *digital phenotyping*, but it is narrower as it “only” relates to the sensing process via mobile technologies such as the smartphone.

It comes not as a surprise that *mobile sensing* and *digital phenotyping* play a growing role in research shedding light on IUD. It has been well-described that humans experience time distortions when using the smartphone [35]. This has likely to do with the data business model behind many applications to be found on smartphones. In particular, social media and Freemium games provide the allowance to use their services in exchange for user data. The exploitation of these digital footprints can result in huge profits when access to digital profiles is sold from social media companies to marketing agencies for microtargeting purposes. Therefore, companies behind social media and Freemium games have a keen interest to design platforms, which are immersive [36]. In other words, the online platforms are designed in a way that users spend more times on the platforms than originally planned. Aside from fostering addictive tendencies towards platform use, this data business model is discussed to be also responsible for endangering democracy and privacy [37]. As many users have problems in assessing their online behavior, methods from *Psychoinformatics* surprisingly can be also useful to counteract Internet Use Disorders—in other words, digital technologies are used to reduce problematic online use.

**Fig. 1:**
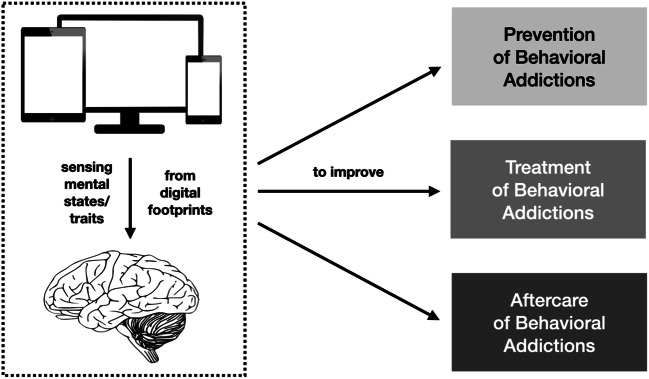
Sensing mental states/traits from digital footprints can be used to improve prevention, treatment, and aftercare of mental disorders including addictive behaviors (please note that the computer/brain symbols in the figure have been taken from pixabay.com and are license free)

The present overview will now touch upon three areas where methods from *Psychoinformatics* can be helpful to improve the understanding and treatment of Internet Use Disorders (see also Fig. 1 on the next page).

## The Potential of Psychoinformatics for Psycho-Diagnostics in the Area of Internet Use Disorders: a View on Prevention

In the previous section, it has already been described that humans face time distortions while using digital technologies. Indeed, it has been shown that certain aspects of smartphone use are over- or underestimated [35, 38], likely also depending on how persons are asked in a survey. Therefore, it becomes apparent that it is very important to also record the problem behavior in the online area of interest to get objective insights into usage patterns of a person. Of importance, self-report will *not* be obsolete. First of all, the near future will need to carve out which set of digital variables on smartphones and other devices are actually linked to addictive online behaviors and also help to track the stages from habit formation to addictive behaviors including its manifold symptoms ranging from loss of control to preoccupation including significant impairments such as loss of a significant relationship or job. Therefore, the digital and self-report data sources need to be brought together. When foreseeing a future where data patterns have been carved out, which map near perfect on each other (and we are a long way from this), from our view, discrepancies between objective recorded behavior on the one side and self-view of a person will still be very interesting to get insights into psychopathologies [35, 39, 40]. Although it is too early to speak of something like “smartphone addiction” (and the term itself needs to be seen very critical in the meanwhile, because people develop addictive behaviors to the apps on the phones and not the phone itself [41, 42]), we would like to revisit some of the *Psychoinformatic*s-oriented research in this field demonstrating its relevance. For instance, an experimental work recording behavior of 64 participants over a time course of 8 weeks demonstrated that high interactions with social media apps are linked to smartphone use disorder/mobile IUD [43], something also observed in another app-tracking study by Shin and Lee [44] (please note that this work mentioned use of entertainment apps not to be a good predictor of problem behavior in the area of smartphone use). A work by Rozgonjuk et al. linked higher problematic smartphone use to more minutes spent on the phone [45], and another work linked objective higher smartphone use to lower academic performance [46]. For a recent review on relevant research, please see the work by Ryding and Kuss [47]. Several findings in this field are also in line with self-report studies showing a strong overlap between social networks and smartphone use disorder [48, 49]. At the moment, we are convinced that both relying on app-tracking for objective recording of online behaviors and asking participants on symptom load related to IUDs (preoccupation with online use, loss of control over online use, significant impairments in everyday life due to online use, etc.) via surveys helps to get the most accurate picture on a person’s tendencies towards IUDs. Such a combination of data might also be best in terms of building an early prevention scheme, alerting the user when he/she is transiting from a habit use towards a problematic use of an application on the phone (such as rising numbers of checking an app). Of note, such an approach becomes more and more feasible with applications readily available for scientists without a computer science background [50••]. This said, all-in-one-solutions are still missing to our knowledge to answer how much a person is—for instance—gaming on different devices such as desktop computer, smartphone, tablet, and so forth. Beyond that, privacy and compliance issues arise from app-tracking, something we discuss in the outlook section.

## The Potential of Psychoinformatics for Intervention-Purposes in Internet Use Disorders

Montag, Reuter, and Markowetz argued in 2015 to implement methods from *Psychoinformatics* to counteract Internet Use Disorders [51, 27]. They suggested that a relevant interventive service would be to endow online users with the opportunity to set a self-chosen maximum time of online use regarding a self-chosen app or a device such as the smartphone [51]. This could be seen as a form of psychoeducation, but from a different perspective also as a way to attract interested persons to participate in a large-scale study assessing smartphone behavior with thousands of users [52]. Ironically, the tech companies from Silicon Valley including Facebook and Apple offer varying tools to help users to better regulate their screen time. While Facebook allows their users on its Facebook and Instagram platform to get notifications after a certain usage time, Apple sends each of their iPhone users a weekly report on their screen time. Although this all sounds positive and indeed could be seen as potential intervention to cut down unnecessary screen time, numbers demonstrating if such an intervention really helps to reduce online time are mostly lacking to the best of our knowledge. The technology companies have not provided the public with numbers on how online time changes in people using the aforementioned services, nor have they provided insights on how online time and well-being changed in Instagram users, when the feature of “Likes” on other users’ profiles have been made invisible [53]. Despite this lack of data, we are optimistic that users can be nudged towards a healthier use of online content by clever system design [36]. For instance, a recent experimental work demonstrated that smartphone and social media use can be significantly reduced by an easy intervention, namely switching from a color mode on the smartphone to a gray scale color mode [54]. Moreover, we are convinced that an improvement of problem behavior with respect to smartphones in general, and social media/gaming more specifically, can be achieved if platforms are designed in a less “persuasive way.” A review by Montag et al. provides a detailed overview on design elements built-in social media platforms and Freemium games together with psychological/economic theories likely prolonging online time [36]. Again, this emerging research field deals with multifaceted problems. Although social media companies and game developers have detailed knowledge about how the design of their platforms impacts on their users (years of AB-testing on huge sample sizes helped them to get this knowledge), independent scientists have only restricted (if at all) access to such data [55]. Much of what we know has been derived by empirical research going the hard way of “reverse engineering,” namely reconstruction of what is happening on these platforms. For a detailed critique, see both the works by Montag and Hegelich [37] and Hegelich [56]. This said, well-conducted *Psychoinformatics* research will be of great help to answer research questions of fundamental importance: How does a digital environment look like which not fosters addictive tendencies towards a platform but rather enhances well-being and meaningful social interaction (hence pronouncing the “social” in “social media”)? A first guideline has been proposed by ind.ie and their ethical design manifesto also being discussed in Montag and Hegelich’s recent work [37].

What has not been discussed so far is the perspective from the clinician’s perspective. Tracking the relevant digital markers on smartphones and other devices covarying with the addictive disorder will help to get insights into the development of the mental disorder and could even result in real-time interventions, when the digital data hint towards an emergency state on the patient side (for exact processes of such an intervention, see an app developed by Mindstrong Health to treat patients with affective disorders as described in Dagum and Montag [57]). In this context, we explicitly mention that this approach not only comprises the study of patients, but the complete spectrum from healthy via problematic to diagnosed psychopathological behavior. Applying such a dimensional approach will likely also help to carve out digital usage patterns (or digital footprints) being uniquely related to different sections of the continuum.

## The Potential of Psychoinformatics for the Area of Aftercare in Internet Use Disorders

After we have discussed the potential of *Psychoinformatics* within the context of prevention and intervention (hence a form of treatment) in the field of IUD, we would like to add some thoughts on the aftercare of IUD. Again, empirical works in this area are scarce. Therefore, we like to discuss some ideas, which will need empirical back up in the near future.

Let us imagine a patient who successfully has overcome a Gaming Disorder. First of all, digital apps could be helpful to restrict access to computer games in the future by also relying on tracking his/her online behavior. Of importance, such tracking should only happen after having received informed consent from the patient as part of the standard procedures carried out before starting a treatment. In our opinion, a tailored approach is very important in the treatment of IUD. With a society being permanently online and requirements of doing jobs in online environments (see also the rise of digitalization due to the COVID pandemic and measures of prevention [58]), it is not reasonable to refrain generally from being online. Therefore, it is of utmost importance to carve out in detail the online problem behavior at hand (is it gaming alone, social media, or a combination of different online contents?) and to work in particular on the problem behavior in the mentioned areas of a patient. Technology can help to support the patient in cutting down the detrimental aspects of his/her online use by restricting it to a defined time limit or in the worst case scenario (and with the approval of the patient) to completely ban it from his/her devices. Filtering of ads related to problem behavior might be also a good way to reduce cue-reactivity, something to be discussed of relevance in many addictive behaviors (for instance, see importance of cue reactivity to understand alcohol use disorder [59]). In general, tracking of online behavior can help in the aftercare scenario (as in early prevention) to monitor if online behavior (re-)escalates in terms of usage. As it is well known that online time alone is not a sufficient criterion to diagnose an IUD, it should be mentioned that different patterns of online use relatively robustly provide insights into negative affect accompanying addictive behavior: Withdrawal from society can be tracked via global position signal data [60] or less social interaction with others via phones and negative emotionality can be inferred from text mining messages [61]. The potential has been also described both to infer mental states in times of crisis such as COVID-19 to guide policy makers [62, 63] and study the development of technological habits during the pandemic.

## Challenges in the Realm of Applying Methods From Psychoinformatics: Statistics and Privacy

The following thoughts are not only relevant for the study of IUDs, but in general when discussing the potential and challenges of *Psychoinformatics*.

(Digital) Big Data, as the essential data basis to be studied in *Psychoinformatics*, can be characterized by the three VVVs: variety, velocity, and volume [24•]. This brings challenges for both psychologists and psychiatrists working in the field of addictive disorders (and other areas of their respective disciplines), relying often on classic inferential statistics. Instead, this new interdisciplinary research endeavor warrants analysis strategies including machine learning, posing itself several problems, such as the problem that is often not clear what the machine actually has learned to provide the researchers with an outcome [64••]. Aside from this, problems arise because Big Data analysis might lead to spurious findings (multiple testing might lead to lots of false-positive findings; therefore, replication of findings is of high relevance—as in other fields of the psychological sciences [65]). Beyond statistical problems, *digital phenotyping* and mobile sensing endangers privacy of the patients afflicted [57]. It needs to be made sure that the recorded data underlies strong data protection plans together with ethical guidelines in what areas what kind of data can be used. For instance, it makes a strong difference if such data would be used to calculate the costs for an insurance policy (which should be prevented by the legislator) or if such data would be used to improve psychodiagnostics, treatment, and aftercare of Internet Use Disorders and related psychopathologies to improve the well-being of a patient. Only when patients know that the data are exclusively used to improve their medical outcome, trust can be established in such technologies. In times of surveillance capitalism [66], this is more important than ever and a work by Montag, Sindermann, and Baumeister [67] discusses prerequisites to anchor *mobile sensing* and *digital phenotyping* as valid, reliable, objective, and fair tools in the psychological and psychiatry sciences: Among others, transparency about what is tracked and what is not tracked for what purpose should be a mandatory part of standard operation procedures before persons opt in for behavioral digital tracking. Beyond this, the scientific community needs to make sure that robust knowledge on digital patterns covarying with the different stages of developing an Internet Use Disorder has been derived from well-carried out clinical trial studies.

## Conclusions and Outlook

The present review aimed to provide insights into a fascinating and rapidly evolving research field posing the question how digital technologies which are the probable cause of manifold online addictive behaviors (driven by the economic data business model) can be used as potent tools to improve prevention, intervention/treatment, and aftercare solutions in the context of IUD. From our observations, most studies in the field currently focus on the psychodiagnostics part linking individual differences in self-reported online addictive behaviors to a myriad of digital footprints with less research efforts in the other mentioned areas of intervention/treatment and aftercare. Clearly, these latter areas are of equal importance and urgently need new insights from empirical works. Please note that we have not discussed the lively field of online therapy—we focused in this work on the potential of *digital phenotyping*/*mobile sensing*. Such a narrow view for sure brings limitations, as it neglects important insights from studies for instance investigating the effectiveness of online therapies [68]. Another important issue to be further discussed relates to the implementation of *digital phenotyping* and *mobile sensing* into traditional intervention approaches in terms of blended treatment [69] as well as models of care. In particular, stepped care models could serve as an approach in which digital interventions would be of special importance in the first less intensive steps as well as in aftercare. Moreover, face-to-face interventions could be combined in all steps with *digital phenotyping* and *mobile sensing* to increase efficacy. To date, such approaches have not been systematically introduced and tested in the field of IUD which is one of the challenges of future research in this area.

## Footnotes

1 https://icd.who.int/browse11/l-m/en#/http://id.who.int/icd/entity/338347362 (accessed on 13th November 2020)
